# The Institutional Library

**Published:** 1915-08-07

**Authors:** 


### THE INSTITUTIONAL LIBRARY.

### The History of the Voluntary Aid Detachments.

### The Men and Women of Kent.

Kent's Care for the Wounded. By Paul Creswick, G. Stanley Pond, and P. H. Ashton. With a Preface by Sir Gilbert Parker, Bart., M.P. (Hodder and Stoughton. 2s. net.)

Even in this age of improvisation in national service no story could show us more clearly the wonderful reserves of strength in the English character than the account of the work of the Voluntary Aid Detachments. The authors of this volume deal with the work of the V.A.D. in one county only—Kent—and, inspiriting as their tale is, yet a similar history could be written of practically every county. Praise can hardly be sufficient for those who, too often in face of discouragement, made such a work possible. The result, as this book sets out to show, is, in a sense, a vindication of the hospital amateur. The history of the movement is well known. The ancient order of St. John of Jerusalem founded what was perhaps the first true hospital, a "Hospice for the Entertainment of Wayfarers and the Reception of the Sick," which dates from the reign of Athelstan. Descended from this are the present day St. John V.A.D.s. The British Red Cross Society, founded by King Edward VII., and placed under the Presidency of Queen Alexandra in 1905, succeeded Lord Wantage's "British National Society for Aid to the Sick and Wounded in War," and the "Central British Red Cross Council," which date respectively from 1870 and 1898. Both societies worked in connection with the Territorial Force Association provided under Lord Haldane's scheme. But the object with which such detachments were initiated should be clearly remembered in order to appreciate to its full extent the adaptability displayed in the past year. They were formed in connection with the Territorial Force only, and the Territorial Force, of course, was founded as an army for use in case of invasion, not, as it has become under pressure of war, an army for foreign service. The V.A.D.s, therefore, had, until the declaration of war last August, the same difficulties to combat as the Territorials in the incredulity of the majority of their countrymen as to the possibility of invasion. Secure as the nation has been in the knowledge of England's immunity from invasion for two hundred years, this security was considered redoubled nowadays by the size and efficiency of the British Fleet. In spite of some inertia, however, a widespread organisation existed; and when, upon the declaration of war, the detachments were called upon they were prepared to justify their faith that they "would one day be needed."

In Kent the three organisations already mentioned at once agreed to amalgamate under one County Director. Dr. Cotton, the Deputy Commissioner of the St. John Ambulance Brigade and County Director of the Territorial Force Association, agreed to fill this position, with Dr. Yolland as his chief of staff. A plan of campaign was formulated and the organisation was placed upon a business basis. Dr. Cotton's health, however, unfortunately broke down under the strain of his duties and he was compelled to resign, but the Earl of Darnley, himself actively interested in hospital work, patriotically offered to continue the campaign, with Dr. Yolland's valuable assistance.

The amount of preparation necessary was, of course, very great. Articles of equipment had to be requisitioned, and here people of every class were quite embarrassing in their willingness to give all that was needed. Only those who undertook this work can realise the generosity shown by the very poorest in supplying the hospitals. A redundance of some articles occurred in different districts, and, to obviate this, central depôts were planned to exchange superfluous goods and to make up deficiencies. Training and lectures also had to be provided, but throughout the county medical officers gladly gave their time to supervising the classes.

Then there was the question of finance. Money was needed to pay for structural alterations necessitated in cases where public buildings, such as halls and schools, were in process of conversion into hospitals. It was foreseen, too, that a supplementary allowance would probably be needed in some cases over and above that allowed by the War Office. A committee was therefore called together, under the presidency of the Marchioness of Camden, and a fund was inaugurated by Mr. J. W. Wheeler-Bennett. £10,000 was soon realised, and the amount is still increasing. Thus it was that, when the order of mobilisation arrived on October 13, 1914, Kent found itself prepared; 10,000 patients have already passed through the wards of the Kent Voluntary Aid Hospitals.

This book, therefore, should be read, both for the stirring tale which it unfolds and for the worthy object with which it is written, for the profits are given to the Kent County War Fund. As we said above, the history of one county organisation is, in essence, that of all; and though the history of each county's work will surely in time be written, the present volume is sufficient to show how worthy of the hospital historian such a subject is. We hope Kent's example will be followed. The work is well and enthusiastically done.

### The Devon V.A.D.

VOLUNTARY AID IN DEVON. Edited by W. FOTHERGILL ROBINSON. (Eland Brothers, High Street, Exeter. 1s net.)

THE story of the development of voluntary aid work in Devon makes very interesting reading in the hundred odd pages allowed to Mr. Fothergill Robinson. A great point is made of the value of the headquarters administration, with its own staff, to which the success of the work in the county is in the main attributed. Begun in 1909, when detachments were organised at Exeter, Torquay, and Plymouth, the local detachments, while allowed considerable scope for initiative, were yet placed under the control of the county headquarters, and the wisdom of this plan is attested by the claim that when " the call came " it was found necessary merely to increase the staff, and not to reorganise it. It seems to have been proved in practice that the theory that the work would be required only in the case of invasion proved readily applicable to the reception of wounded from the Front, and to the care of troops quartered in the county. The Exeter, Newton Abbot, and Torquay hospitals receive their quota of patients direct from the hospital ships on arrival at Southampton, and fifteen smaller institutions are devoted to the sick among the local troops, and to such men as have been discharged from the military hospitals. Each hospital was at once placed under medical control, and a great point is made of the fact that volunteer women were placed as probationers under the control of a trained staff. The book is valuable not merely because the story of the work is well told, but because the grouping of the chapters, under such headings as "Headquarters," "Work of the Hospitals," "Finance," and "Catering at Rest Stations," provides the reader with a bird's-eye view not merely of the work, but of the actual working and system of organisation. An account is then given of the actual institutions, and the planning of the work is exceedingly good. In its double capacity as a moving story and a handbook of organisation it deserves a wide sale, for its interest will not die when the war is over.

### A Tuberculin Guide.

A GUIDE TO THE USE OF TUBERCULIN. By A. W. R. COCHRANE, F.R.C.S. Eng., and C. A. SPRAWSON, M.D. Lond., M.R.C.P. (London: John Bale, Sons and Danielsson, Ltd. 1915. Pp. 180. Price 5s.)

IF ever there was a drug which needed a guide to its use, that drug is surely tuberculin. The fact cannot be disguised that lives have been shortened through its use either by enthusiasts or ignoramuses, and no inconsiderable element of charlatanism has sheltered under protection of this once too popular form of treatment. To foster a deeper respect for the potentiality of the drug is a praiseworthy attitude, and even to-day to encourage an unprejudiced scepticism regarding the "results" obtained by tuberculin therapy is the wisest course both from the point of view of the practitioner and of his patient. In the hands of honest experts—a small band—tuberculin has justified itself in a limited class of case. But the administration of this drug is far more widely spread, and the need for a guide is thus urgent. Majors Sprawson and Cochrane, of the I.M.S., are both experienced in the treatment of pulmonary tuberculosis, and they now offer a guide to the use of tuberculin which has much to recommend it. It attempts to give more definite instructions than are usually to be found in larger text-books, and is thus calculated to be of practical help to the medical man in charge of a case. The somewhat confusing nomenclature is well tabulated, and the method of preparing the dilutions is adequately described. A whole chapter is devoted to the consideration of a working hypothesis of the action of tuberculin. Here the authors deal ably with a difficult subject, and have produced not only an interesting summary, but also as helpful a series of explanations as could be desired, having regard to the state of our knowledge on these points. Succeeding chapters describe the general principles of dosage, the proper interpretation of reactions, and the conduct of a full " course " of the drug.

The authors' methods will be recognised as based on careful principles, though there is shown a tendency to recommend the use of tuberculin in cases which might fairly be considered by many English authorities as unsuitable for even the most cautious doses. This section, with its numerous accompanying charts, is one to be thoroughly studied by any tuberculosis officer who intends to use this adjunct to the treatment of pulmonary tuberculosis. To be guided by the authors of this manual is at least to have some principles to go upon, and the strict observance of all the "rules" should prevent harm being done to any patient. A survey of the contra-indications of tuberculin treatment makes a practical chapter; and on the inclusion of one of these contra-indications the authors may be congratulated—namely, when the patient will not be under the charge of one physician for more than three or four weeks. In spite of the now considerable statistics and personal observations available regarding series of cases treated with tuberculin, there is not yet any definite consensus on the subject. But the great extension in the use of this remedy, both at home and on the Continent, suggests that the drug is capable of favourably influencing the course of the disease, if not materially affecting the ultimate prognosis. Therefore to this concise and lucid guide a cordial welcome may be extended, with the hope that rash experiments may henceforth be replaced by judicious and wary administration.

### A List of War Help Societies.

WAR DISTRESS AND WAR HELP: Short Catalogue of the Leading War Help Societies, showing their Scope and the Addresses of their Offices. By HELEN DONALD-SMITH. (John Murray. Price 6d. net.)

Now that the inevitable cry of overlapping in the work and number of the various war charities is making itself heard, a warm welcome should be given to Miss Helen Donald-Smith's pamphlet. It comprises in thirty-four pages a catalogue of War Help Societies, which are given in alphabetical order. The particular work of each is given in a sentence, which is followed by the address of the office, from which further particulars may be obtained. In case it may be suggested that the scope of the pamphlet is too small, and that within its brief compass many associations must be omitted, it is fair to give the author's explanation. "Besides the general associations given in the catalogue, however,"

(*Continued on p.* vi.)

